# Inhibition of *Escherichia coli* invasion into bovine mammary epithelial cells previously infected by *Mycobacterium avium* subsp. *paratuberculosis*

**DOI:** 10.1080/01652176.2020.1716278

**Published:** 2020-01-28

**Authors:** David Germano G. Schwarz, Junnia L. Pena, Isabel A. Carvalho, Abelardo Silva Júnior, Maria Aparecida S. Moreira

**Affiliations:** a Veterinary Medicine, Universidade Federal do Piauí (UFPI), Campus Cinobelina Elvas (CPCE), Bom Jesus, PI, Brazil; b Departament of Veterinary, Sector of Veterinary Medicine and Public Health, Universidade Federal de Viçosa (UFV), Viçosa, MG, Brazil; c Department of Pathology, Universidade Estadual do Maranhão, São Luís, MA, Brazil

**Keywords:** Bovine, cow, ex vivo, coinfection, mastitis, *paratuberculosis*, epithelial cells, mammary gland

## Abstract

**Background:**

The coinfection process of *Escherichia coli*, an etiological agent of clinical mastitis and *Mycobacterium avium* subsp. *paratuberculosis* (MAP), a non-mastitic etiological agent in the bovine mammary gland is not fully known.

**Objective:**

Verify the ability of MAP to interfere with the invasion and translocation of *E. coli* in bovine mammary epithelial cell line (MAC-T).

**Methods:**

For the invasion assay, MAC-T cells were challenged with MAP K10 for 2 h and then challenged with *E. coli* for 10, 30 and 120 min. For the translocation assay, the trans well plates were used and the challenge sequence was repeated as previously described. The amount of *E. coli* in the assays was determined by counting colony forming units (CFU) in Luria-Bertani medium. Quantitative real-time PCR was used to quantify MAP in MAC-T cells. To verify the viability of the MAC-T cells, the MTT assay was performed. MAP culture supernatant was also evaluated at different percentages for *E. coli* growth.

**Results:**

Previous MAP infection in MAC-T cells inhibited *E. coli* invasion in 10, 30 and 120 min. No significant interference of MAP in the translocation of *E. coli* from the apical-basal direction was verified. Quantity of MAP DNA inside the MAC-T cells was statistically similar. Neither reduction in MAC-T cells viability was detected during the experiment nor MAP-released factor in the supernatant inhibited *E. coli* invasion.

**Conclusion:**

These findings suggest that MAP-positive cows could be more resistant to *E. coli* infection, but when infected, could rapidly translocate *E. coli* to the subepithelial region.

## 1. Introduction

Coinfection is a complex interaction process between microorganisms and the host cell. Different factors related to infection route, target cell, type of pathogens, intensity of the immune and inflammatory response are important to the success of the coinfection process. In mastitis, an inflammation of the mammary gland, the mammary epithelial cells are the first barrier that interacts with different pathogens in an attempt to avoid infection and inflammation. In bovine mastitis, among more than 150 different etiologic agents, *Escherichia coli* is the main bacteria isolated from environmental mastitis (Bradley 2002). This infection causes an intense immune response which elicits bacterial clearance in a short time. Although the mastitis caused by *E. coli* is considered a transient infection, it can advance to a clonal persistent intramammary infection, suggesting an adaptation of this pathogen to the bovine udder environment (Bradley and Green 2001; Dogan et al. 2006). Invasion and persistence of *E. coli* inside of mammary epithelial cells is not fully understood and there are currently conflicting hypotheses that seek to explain these process. Dogan et al. (Dogan et al. 2006) verified that *E. coli* can use the rearrangement of the cytoskeleton and phosphorylation-mediated signaling cascades to facilitate invasion process. However, Passey et al. (2008) identified that it occurred though a membrane-bound endocytic vacuole. Otherwise, *Mycobacterium avium* subsp. *paratuberculosis* (MAP) can infect bovine mammary epithelial cells and be eliminated by milk, although its contribution to the inflammatory process of the mammary gland is not yet clear. MAP is the etiologic agent of paratuberculosis, characterized by chronic and incurable granulomatous enteritis in ruminants, with reduction in milk production, diarrhea, progressive weight loss, malnutrition and death (Collins 2003; Lombard 2011). In addition, the lesion characteristics present in some human intestinal inflammatory diseases, such as Crohn’s disease (CD), and the presence of MAP in intestinal human biopsies, suggests a possible relationship between this agent and CD. Once ingested, MAP enters the organism’s digestive tract through Peyer’s patches by fibronectin-dependent mechanisms or by endocytosis in the enterocytes (Pott et al. 2009; Bermudez et al. 2010). It can then reach the subepithelial macrophages, the blood stream and multiply in satellite lymph nodes (Bermudez et al. 2010). Furthermore, MAP is able to infect bovine mammary epithelial cells by both, basal and apical surface efficiently and persists inside vacuoles (Patel et al. 2006; Lamont et al. 2012), similarly as described by Passey et al. (2008) to the *E. coli* infection. Thus, MAP could reach the mammary epithelial cells via the systemic route (Lamont et al. 2012) or through the upward contamination of the teat canal (Schwarz et al. 2017). Although there is no evidence that MAP is associated with mastitis (Larsen and Miller 1978), a study by Wilson et al. (Wilson et al. 1993) verified that cows infected with MAP had a reduction in cases of subclinical mastitis caused by *Staphylococcus aureus* and *Serratia* sp., suggesting a possible relationship between mastitis and paratuberculosis.

Coinfection study of MAP and *E. coli* isolated from bovine mastitis was recently described by Schwarz et al. (2018). In this study, the previous *E. coli* infection of bovine mammary epithelial cell line (MAC-T) increased a rapid baso-apical translocation of MAP to the supernatant of the cells. These results demonstrated that a mammary epithelial cell previously infected by a mastitis-associated bacterium facilitated the subsequent infectious process by another non mastitis-associated bacterium. Others also have demonstrated the influence of coinfection between pathogenic bacteria in the mammary gland (Bouchard et al. 2013; Assis et al. 2015; Li et al. 2016). Probiotics (*Lactobacillus casei* and *Lactococcus lactis* V7) were able to inhibit the adhesion and invasion of *Staphylococcus aureus* and *E. coli* in mammary cells (Bouchard et al. 2013; Assis et al. 2015). Similarly, the presence of cell wall components from *Saccharomyces cerevisiae* were effective in inhibiting MAP adhesion to MAC-T and bovine primary epithelial cells (Li et al. 2016).

The aim of this work was to verify whether MAP-infected bovine mammary epithelial cells interfere with a subsequent internalization and translocation of *E. coli* strain isolated from a cow with mastitis in an ex vivo model.

## 2. Materials and methods

### 2.1. Ethics statement

All experiments were carried out in accordance with the Ethics Committee on Animal Use (CEUA) at the Universidade Federal de Viçosa, Brazil, under approval protocol 36/2014.

### 2.2. Bacterial strains and culture

MAP K-10 standard strain and an *Escherichia coli* strain (Schwarz et al. 2018) isolated from milk from a case of bovine clinical mastitis (kindly provided by Dr. Srinand Sreevatsan from the Laboratory of Udder Health, Minnesota Veterinary Diagnostic Laboratory, University of Minnesota, Saint Paul) were used in this experiment. Culture of MAP- K10 was tested for purity using IS*900* PCR and cultivation in Middlebrook 7H9 agar medium supplemented with 10% glycerol, 1% oleic acid-albumin-dextrose (OADC), and mycobactin J (2 mg/L) (Allied Monitor Inc., Fayette, MO, USA). To ensure that the isolated bacteria were *E. coli,* DNA were extracted by the Promega® Kit (Promega, Madison, WI, USA), following the manufacturer’s protocol. The *uspa* gene target was amplified by PCR and the amplified fragments were sent to Macrogen Corporation (Seoul, South Korea) for sequencing and revealed 99% similarity with the *Escherichia coli* strain. In addition, biochemical tests were performed: catalase test (positive), oxidase test (negative), Triple Sugar Iron Agar test (negative), methyl red test (positive), citrate test (negative) and lactose fermentation test (positive), confirming that it was an *E. coli* strain.

The MAP strain was grown in Middlebrook (MB) 7H9 broth containing 0.2% glycerol, 10% OADC and mycobactin *J* (2 mg/L) (Allied Monitor Inc., Fayette, MO, USA), at 37 °C for six weeks until reaching the optical density (OD_600_) of 0.5 (equivalent to 10^6^ CFU/mL). The culture was determined free of contaminating microorganisms by the absence of colonies in Brain Heart Infusion (BHI) medium. *E. coli* isolate was grown on Luria-Bertani (LB) agar overnight and one colony was incubated for 4 h (to mid-log phase) at 37 °C in 5 ml BHI broth without shaking to an optical density at 600 nm [OD_600_] = 0.3 (equivalent to 10^8^ CFU/mL).

### 2.3 Mammary epithelial cells and culture conditions

Bovine mammary epithelial cells (MAC-T) line have been widely used in adhesion/invasion assays (Li et al. 2016) and thus was used in this experiment. MAC-T cells (kindly provided by Dr Yung-Fu Chang, Cornell University, Ithaca, USA) were cultured in T25 cell culture flasks (TPP Techno Plastic, St. Louis, MO, USA) at 37 °C with 5% CO_2,_ containing Dulbeccòs Modified Eagle Medium (DMEM; Gibco-BRL, Grand Island, NY, USA) supplemented with 10% heat inactivated fetal bovine serum (FBS; Sigma-Aldrich, St. Louis MO, USA) and 1% penicillin/streptomycin (100 μg/ml) up to the confluence of the cells. For the experiment MAC-T cells low passage number were used.

Confluency was assessed by visualization under phase-contrast microscopy. Integrity of the monolayer was determined by the following methods: (i) phase contrast microscopy observation, (ii) staining of monolayer by crystal violet and (iii) trypan blue (0.25%) permeability assay (optical density at 540 nm), where the trypan blue was added to the monolayer and 1 h later the supernatant of the lower chamber was obtained for spectrophotometric reading, as previously described (Patel et al. 2006). After visualizing the monolayer on a Nikon phase contrast microscope (Nippon Kogaku K. K., Tokyo, Japan), cells were washed with PBS (pH 7.2), resuspended by the addition of 0.25% trypsin (Sigma-Aldrich, St. Louis, MO, USA) and subsequently used in the experiments.

### 2.4. Internalization assay

Internalization assay was performed following the procedure described previously by Bouchard et al. (2013) with modifications. Briefly, confluent monolayer of MAC-T cells, prepared as described above, were washed twice with PBS, dislodged with 0.25% trypsin for 5 min, and counted with 0.4% trypan blue (Invitrogen, Eugene, OR, USA) using a hemocytometer chamber slide. MAC-T cells were seeded (3x10^5^ cells) into 24-well polystyrene plates within cell culture medium (DMEM, 10%FBS, without antibiotics) and incubated in a humidified chamber at 37 °C with 5% CO_2_ for 24 h. After 90-95% of monolayer confluence, MAC-T cells were washed twice with warmed PBS and challenged with MAP (1.0 x 10^6^ CFU) for 2 h. Before MAP challenge, 21-gauge syringe needle was used to create a single cell suspension of MAP and upper three-fourths of it were used for the challenge. Then, MAC-T cells were washed three times with warmed PBS (37 °C) and challenged with *E. coli* (1.0 x 10^8^ CFU/ml) for 10 min, 30 min and 120 min, following an additional 2 h incubation step with DMEM supplemented with gentamicin (100 µg/mL). This step ensured that any extracellular bacteria (adhered or planktonic) did not remain viable due to the bactericidal action of the antimicrobial. Subsequently, culture supernatants were removed and monolayers were washed four times with PBS and lysed with 50 μL of 0.25% (wt/vol) trypsin (Sigma-Aldrich, St. Louis, MO, USA) and 0.1% (vol/vol) Triton X-100 (Amersham, Arlington Heights, IL, USA) in PBS for 10 min. Lysates were serially diluted and plated on LB agar, and bacterial concentrations were determined from the colony counts after incubation at 37 °C for 24 h. Positive control-wells (*E. coli*) and negative control-wells (MAC-T cells) were cultivated as described above in triplicate. All assays were repeated three times.

To verify if MAP was internalized in MAC-T cells previously to *E. coli* challenge, monolayers of MAC-T cells were challenged by MAP (1.0 x 10^6^ CFU) for 2 h plus 10, 30 or 120 min at the same experimental conditions described above. After incubation, monolayers were washed three times with warmed PBS and then treated with 200 µg/ml of amikacin for 2 h at 37 °C to kill extracellular bacteria, as described by Patel et al. (2006). Then, the supernatant was removed and MAC-T cells were washed three times with warmed PBS. After that, wells were treated with 50 μL of 0.25% (wt/vol) trypsin for 10 min and subsequently added 150 mL of DMEM with 10% FBS and used for quantification of MAP DNA; or treated with 50 μL of 0.25% (wt/vol) trypsin and 0.1% (vol/vol) Triton X-100 in PBS for 10 min for culture of MAP. Lysates were serially diluted (10^°^, 10^−1^, 10^−2^ and 10^−3^) and plated on Middlebrook (MB) 7H9 agar containing mycobactin *J* (2 mg/L), 0.2% glycerol and 10% OADC (vol/vol). Bacterial concentrations were determined from the colony counts after incubation at 37 °C for approximately 12 weeks. The assays were performed in duplicate.

### 2.5. DNA extraction and quantitative real-time PCR

Lysates of MAC-T cells challenged only by MAP at different times (2 h plus 10, 30 and 120 min) were used for quantification of MAP DNA. For DNA extraction, the DNeasy® UltraClean® Microbial Kit (Qiagen, Hilden, Germany) was used. All the procedures followed the manufacturer’s instruction. Briefly, 200 µL of suspended cell solution was added in collect tube and centrifuged at 12,000 x g for 30 s. The supernatant was discarded and the pellet was treated with 200 µL of 2% lysozyme and 20 µL of proteinase K (Promega, Madison, WI, USA). Then, for MAP cell wall rupture solution SL was added and vortexed for 5 min. All subsequent steps followed the manufacturer’s recommendations. Finally, the DNA was resuspended in 30 µL of nuclease-free water to column and incubated at room temperature for 2 min.

Quantitative real-time PCR (qPCR) was performed using bactotype MAP PCR Kit (Qiagen, Hilden, Germany) in a Rotor-Gene® Q (Qiagen) real-time PCR Detection System and Rotor-Gene Q Software 2.3. The bactotype MAP PCR Kit is highly sensitive (97%) and specific (100%), with efficiency of 98.6% for detection of MAP DNA. It includes a MasterMix solution composed of specific primers, enzymes and probes. All procedures were followed as recommended by the manufacturer. The total PCR reaction was 25 μL with 17 μL of MasterMix and 8 μL of DNA (template). For quantification, the following relation (available from the manufacturer) was used: 8 µL of the positive control corresponds to 200 copies = approximately 12 genome of MAP. Therefore, the MAP genome number was obtained by relating the Cts of the positive control and the samples. The assays were carried out in triplicate and repeated two times.

### 2.6. Transepithelial bacterial translocation assay

Approximately 3.0 x 10^5^ MAC-T were seeded onto the apical side of 3.0-µm-pore-size Transwell®-Clear inserts (Corning, Lowell, MA, USA) in DMEM containing 10% FBS and incubated at 37 °C in a humidified chamber containing 5% CO_2_ up to the confluence of the monolayer (∼ 2 days). Cell monolayer integrity was verified as previously described (Patel et al. 2006; Schwarz et al. 2018). MAP culture (1.0 x 10^6^ CFU/mL) was pelleted at 12,000 x *g* for 15 min, washed two times in PBS and resuspended in DMEM containing 10% FBS. MAP suspension was then passed through a sterile 21-gauge needle 20 times to create a single cell suspension and upper three-fourths of solution were used for infection. MAP was applied to the upper chamber and allowed to infect the MAC-T cells for 2 h at 37 °C in a humidified chamber containing 5% CO_2_ followed by three washing cycles with PBS. Then, *E. coli* (1.0 x 10^8^ CFU) was applied to the upper chamber and incubated under the same conditions described above for 10, 30 and 120 min. After these times, the supernatant from the lower chamber was centrifuged at 12,000 x *g* for 5 min, resuspended in 100 µL of 0.9% saline solution, serially diluted, and plated onto LB agar for colony count. The negative control was unchallenged MAC-T cells, and positive control was MAC-T cells challenged with *E. coli* only. All assays were carried out in triplicate and repeated three times.

### 2.7. MTT cell viability assay

For the viability assay, MAC-T cells (1.0 x 10^5^ cells) were cultivated in 96-well polystyrene plates, at the same conditions described above, and challenged with MAP (2.0 x 10^5^ CFU) for 2 h. After washing three times with PBS, the MAC-T cells were challenged with *E. coli* (2.0 x 10^7^ CFU) for 10, 30 and 120 min, as described above. After incubation steps, cells were washed four times and incubated in 0.5 mg/mL (3-(4,5-Dimethylthiazol-2-yl)-2,5-Diphenyltetrazolium Bromide (MTT) (Sigma-Aldrich, St. Louis, MO, USA) in PBS for 2 h at 37 °C in 5% CO_2_. The supernatant was removed and 200 µL of Dimethyl Sulfoxide (DMSO) was added for 20 min. The purple color thus formed was measured at 550 nm in spectrophotometer. Uninfected cells were used as negative control (100% viability), and cells treated with 0.1% (vol/vol) Triton X-100 served as a positive control of mortality (0% viability). Relative viability was expressed with regard to uninfected cells. The assays were carried out in triplicate and repeated three times.

### 2.8. Bacterial growth curve assay

MAP culture (OD_600_=1.0) was centrifuged at 12,000 x *g* for 5 min and the supernatant was reserved. Aliquots of 1 mL (1.0 x 10^7^ CFU) of *E. coli* culture (OD_600_=0.1) were centrifuged at 12,000 x *g* for 5 min and the supernatant was discarded. The remaining pellets were resuspended in different amounts of MAP supernatants (100%, 75%, 50%, 25% and 0%) and the proportion of each amount completed with LB broth to 1 mL, and homogenized. Subsequently, 250 µL of each concentration was added in 96 wells plates and every 30 min during 24 h, the bacterial growth rates were evaluated by optical density 600 nm (λ) in automatic spectrophotometer (Multiskan GO, Thermo Scientific, Waltham, MA, USA). For the negative control, MAP supernatant at different concentrations as described above was used without the presence of *E. coli*. For the positive control, different amounts of MB7H9 broth (100%, 75%, 50%, 25% and 0%) were similarly homogenized with LB broth to a final volume equal at 1 mL with *E. coli*. The results were obtained by subtracting the values from the experiment using the MAP supernatant and the positive and negative controls. The pH of the supernatants was measured by the PH meter mPA 210 (Tecnopon). All the experiments were carried out in triplicate and repeated three times.

### 2.9. Statistical analysis

The results obtained were analyzed by two-way analysis of variance (ANOVA) with the Tukey test in Graphpad Prism software (Software, La Jolla, CA). *P* values of less than 0.05 were considered statistically significant.

## 3. Results

### 3.1. Influence of MAP on the internalization of E. coli in MAC-T cells

**Figure 1.**
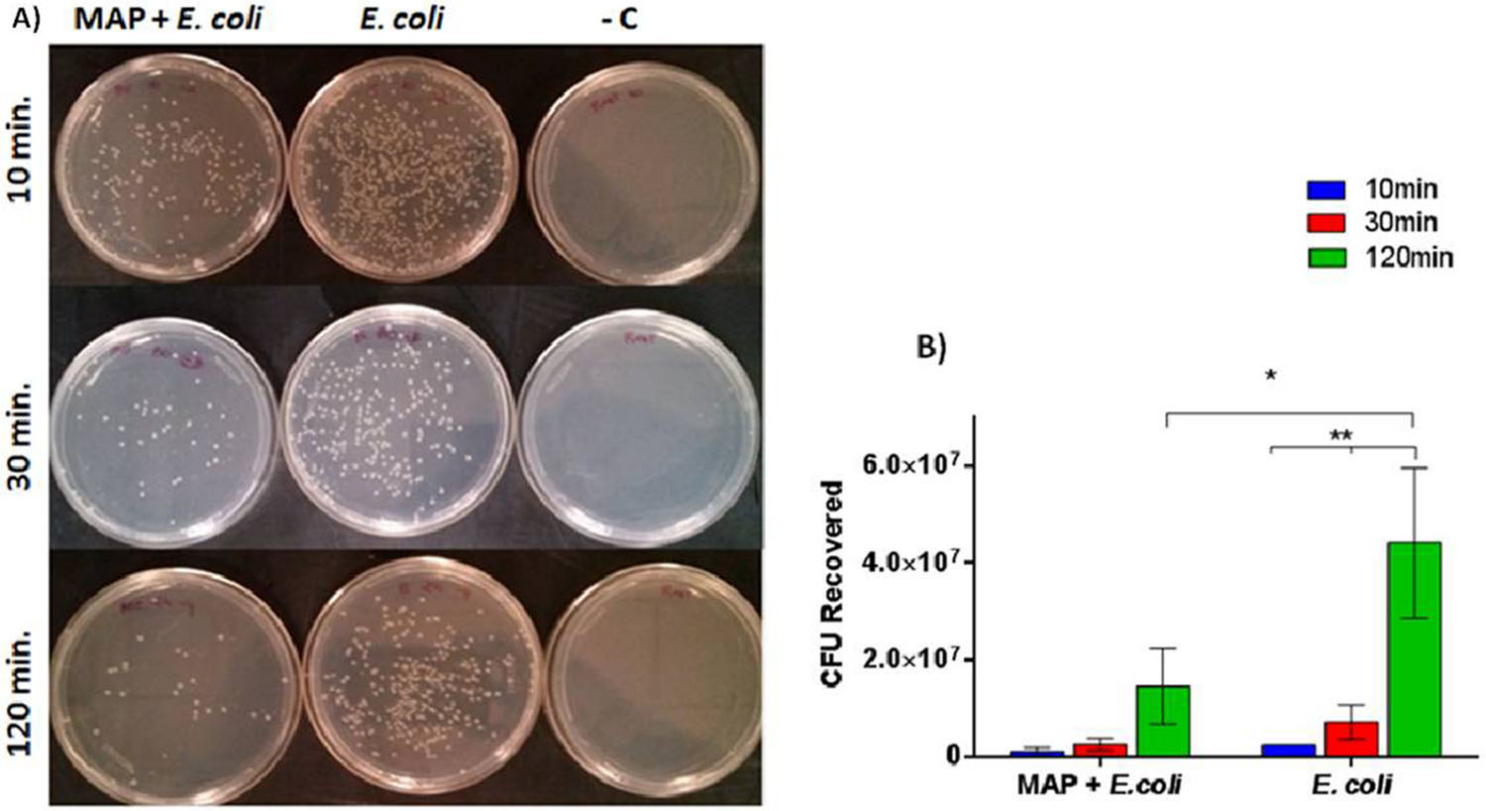
*Mycobacterium avium* subsp. *paratuberculosis* (MAP) in bovine mammary epithelial cell line (MAC-T) inhibits *E. coli* internalization. **(**A) Plaqueation in Luria-Bertani medium (LB) of MAC-T cell lysates challenged by MAP+*E. coli*, *E. coli* and negative control (- C) at different times post-infection (p.i.). (B) Mean and standard deviation of Colony Forming Units (CFU/mL) in different treatments, according to the time p.i. Differences between treatments (MAP+ *E. coli* and *E. coli*) was statistically significant (*P < 0.05). At 120 min, it was statistically significant (**P < 0.01) in relation to the other times analyzed. The assay was carried out in triplicate and repeated three times.

The internalization ability of *E. coli* isolated from bovine mastitic milk in MAC-T cells previously exposure/infected by MAP K-10 was tested in vitro. In Figure 1, a significant (*P* = 0.0307) lower number of *E. coli* colonies was observed (CFU recovered) in cells previously infected by MAP in relation to those without prior infection regardless of the challenge time. The efficiencies of internalization at 10 min, 30 min and 120 min time points were comparable between the treatments (MAP + *E. coli* and *E. coli*). However, no significant differences were found between 10 min and 30 min post infection, but there was a significant difference (*P* = 0.0033) at 120 min post infection.

**Figure 2.**
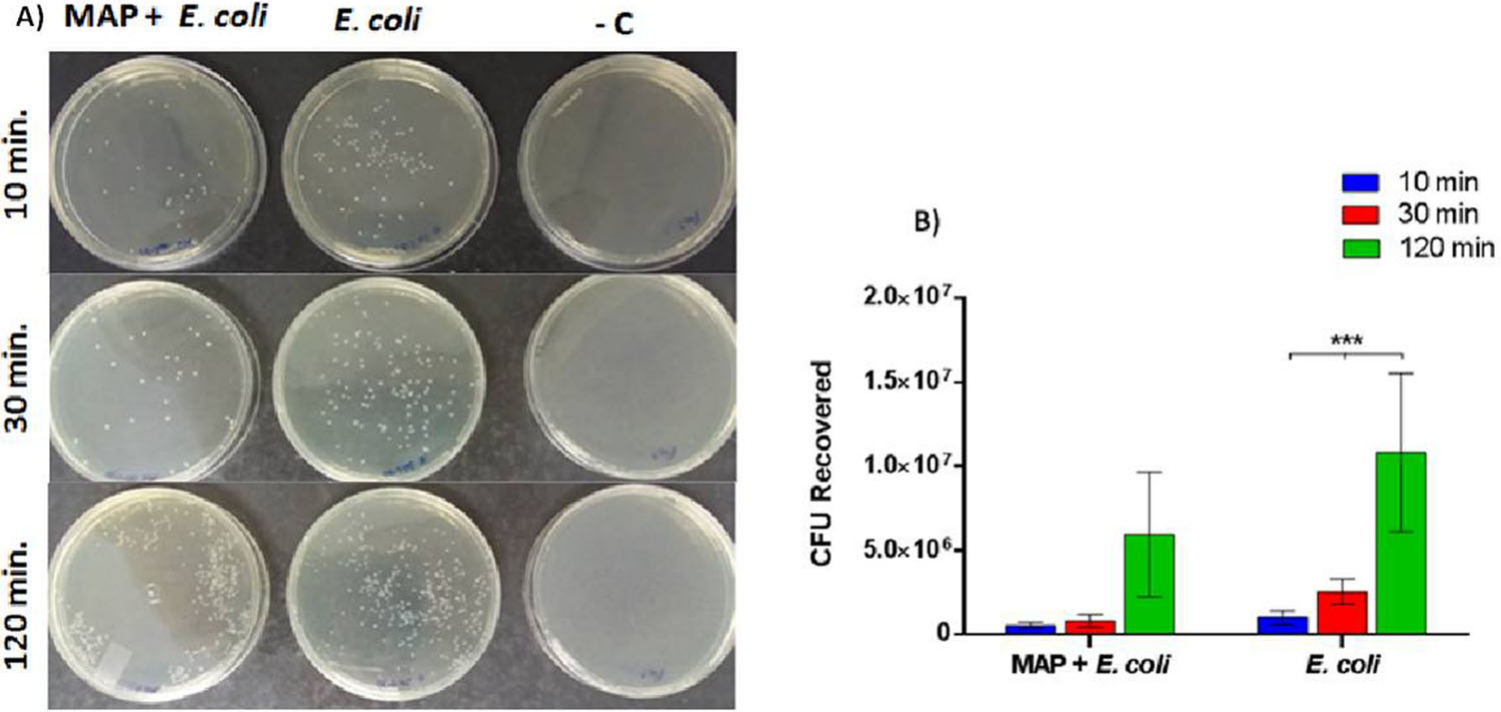
Presence of *Mycobacterium avium* subsp. *paratuberculosis* (MAP) in bovine mammary epithelial cells did not alter *E. coli* translocation. **(**A) Plaqueation in Luria-Bertani medium (LB) of MAC-T cell lysates challenged by MAP+*E. coli*; *E. coli* and negative control at different time post-infection (p.i). (B) Mean and standard deviation of Colony Forming Units (CFU) recovered in different treatments, according to the time p.i. No difference between the treatments (MAP+ *E. coli* and *E. coli*) was observed. In *E. coli* treatment, at 120 min, the translocation was significant (***P < 0.001), regardless of the time p.i. The assay was carried out in triplicate and repeated three times.

In contrast to the internalization assay, there was no statistical difference in translocation assay between the treatments for the 10 min, 30 min and 120 min time points. However, *E. coli* passed through the cells more efficiently at 120 minutes (*P* = 0.0003) when the MAC-T cells were not previously treated with MAP, regardless of the time p.i. or the treatment evaluated (Figure 2).

For the quantification of the genome of MAP in MAC-T cells at 2 h of incubation plus 10, 30 and 120 min of challenge, no statistical differences were observed (*P* = 0.069), detecting 113.3 copies (Ct = 16.5), 128.8 copies (Ct = 19.3) and 111.5 copies (Ct = 16.7), respectively. For the culture of MAP in MB7H9, only one plate at 30 min p.i (10^°^ dilution) growth MAP colonies, with 56 colony forming units (CFU). Other plates were contaminated and were discarded before of 12 weeks of culture time.

### 3.2. Viability of MAC-T cells when infected by MAP and/or E. coli

We showed that the prior infection by MAP significantly reduces the internalization of *E. coli* at 120 min in MAC-T cells (Figure 1). Thus, additional tests were done to investigate the effect of MAP, *E. coli* or MAP + *E. coli* on cell viability. The cell mortality as assessed by MTT assay revealed no difference between treatments, i.e., the presence of MAP or *E. coli* or MAP + *E. coli* does not interfere in the viability of MAC-T cells during 2 h of evaluation.

### 3.3 Interference of MAP supernatant in E. coli growth

To ascertain whether the MAP culture supernatant could interfere with the bacterial growth of *E. coli*, decreasing amounts of the MAP supernatant were homogenized with increasing amounts of LB and the results compared with the positive control. Although 100% of the supernatant allowed considerable growth of *E. coli* during the 24 h of analysis, only with 75% was there a significant difference (*P* = 0.032) in relation to the other proportions. In addition, a higher log-phase of *E. coli* was observed in 75% supernatant compared with other supernatant concentrations. There were no large variations in pH, remaining around 7.2. This demonstrates that pH did not interfere in this experiment.

## 4. Discussion

Among the bacteria that infect the mammary gland, *E. coli* is well known for causing rapid clinical manifestation with an intense immune response (Bradley and Green 2001). On the other hand, *Mycobacterium avium* subsp. *paratuberculosis* (MAP) even being released by milk, appear to poorly stimulate the local inflammatory response and is not considered as the etiologic agent of classic mastitis (Larsen and Miller 1978). Recently, a study showed that cows with *E. coli* mastitis potentially can attract MAP from distant sites and release it into milk more efficiently when compared with cows without mastitis (Schwarz et al. 2018). However, the mechanisms of how a non-mastitic pathogen can infect the mammary gland and interfere in a subsequent infection by another mastitic pathogen are still unknown. We hypothesized that prior MAP exposure/infection in bovine mammary epithelial cells could alter the subsequent ability of *E. coli* to invade these cells at 10 min, 30 min and 120 min. These times where determined since it was verified that MAP K-10 infection in MAC-T cells resulted in early phagosome acidification within 10 min p.i. and reached peak at 30 min (Lamont et al. 2012). Our results demonstrated that MAP was able to rapidly inhibit the invasion of *E. coli* into MAC-T cells. Immediately at 10 min, a reduction in the number of *E. coli* colonies in MAC-T cells previously infected by MAP was observed when compared to cells infected only by *E. coli*. This pattern of infection was observed at all time-points within a 2 h period. Regardless of the time evaluated, rates of internalization into MAC-T cells were 2-to-3-fold lower for cells infected by MAP + *E. coli* than for infected by *E. coli* only, demonstrating that the presence of MAP inhibited this invasion process. These results corroborate with the epidemiological data verified by Wilson et al. (Wilson et al. 1993), where cows positive for MAP had fewer cases of recurrent or chronic mastitis and a low count of infective bacteria. Similarly, it was found that *Lactococcus lactis* V7 was able to inhibit the internalization of *Staphylococcus aureus* and *E. coli* in MAC-T cells, ranging from 45% to 88% inhibition (Bouchard et al. 2013). Besides the presence of bacteria, it was verified that other factors may influence the process of invasion in mammary cells. Silva et al. (2014) verified that the subinhibitory concentrations of antimicrobials in the medium increased the internalization capacity of *E. coli* isolated from bovine mastitis in MAC-T cells, but did not alter the adhesion. This fact suggests that the internalization process is not necessarily associated with the adherence ability. In the current study, the presence of MAP within MAC-T cells was verified by quantification of MAP DNA by qRT-PCR and culture of MAP on MB7H9 agar medium. The number of MAP copies during the post-infection times (10, 30 and 120 min) were found to be statistically the same, demonstrating that the number of MAP in MAC-T cells remained constant throughout the assay. In addition, although colony growth was not verified at all postinfection times, colony-forming unit counts at 30 min p.i demonstrated the presence of viable MAP inside of MAC-T cells.

To evaluate whether the presence of MAP inhibits not only the invasion but also interferes with *E. coli* translocation through the MAC-T cells, a transepithelial assay was performed. Interestingly, the number of translocated colonies of the apical-basal region were statistically the same to MAP + *E. coli* and *E. coli* only, regardless of the treatment. Since MAP inhibited the internalization of *E. coli* but maintained the number of bacteria evaded from the cells when compared to its positive control (*E. coli* only), it could be inferred that MAP enabled higher efficiency in translocating *E. coli* to basal direction. This event could provide a distinct survival advantage that allows the pathogen that invade cells previously infected by another microorganism to escape rapidly from the cells in order to avoid possible changes in the cellular microenvironment, that might impair their survival inside the cell. It has been reported that in non-phagocytic cells, both pathogenic (García-Pérez et al. 2003) and non-pathogenic (García-Pérez et al. 2008) mycobacteria use macropinocitosis with membrane ruffle formation to internalize. Thus, it is suggested that in mammary epithelial cells, MAP could use this mechanism to be internalized in the endosomes. Interestingly, contrary to what is observed in other mycobacteria, when invading mammary epithelial cells, MAP is able to induce phagosome acidification between 10 and 30 min post infection and IL-1β production in order to attract subepithelial macrophages at the site of infection (Lamont et al. 2012). Instead, Passey et al. (2008) have verified that *E. coli* can invade mammary cells without resulting in a visible rearrangement of the cytoskeleton and may activate its intracellular traffic avoiding mechanisms of acidification of phagosomes. Possibly the process of induction and control of intracellular acidification triggered by MAP may have been crucial for the ability of *E. coli* to internalize and/or evade the cells.

A potential concern about the preceding observations was that the reduction of *E. coli* internalization to epithelial cells was due to decreased cell viability. Li et al. (2016) demonstrated that the yeast cell wall components reduced MAP binding to mammary epithelial cells, but this fact was partially attributed to decreased cell viability. Although some authors have worked with the MAP invasion process in MAC-T cells (Li et al. 2016; Schwarz et al. 2018), it is known that MAP is capable of inducing apoptosis in macrophages by a caspase-dependent or caspase-independent mechanism and mitochondrial damage (Periasamy et al. 2013). In the present study, regardless of the time of exposure and the treatment (MAP and *E.coli*, MAP or *E. coli*) there was no reduction in viability of MAC-T cells. These results demonstrate that cell viability did not influence the low *E. coli* internalization.

It was further investigated if the inhibition of *E. coli* internalization in MAP-infected cells could be related to some MAP-released factors (e.g., proteins) that could decrease *E. coli* growth or metabolism. The higher efficiency of *E. coli* growth in the proportions of 75% and 100% of MAP supernatant suggested that MAP can release substances capable of stimulating the growth rate of *E. coli* isolated from bovine mastitic milk. It has been reported that high concentrations of reactive oxygen species (ROS) impair *E. coli* growth due to irreversible damage to cellular components (Imlay 2008; Baez and Shiloach 2013). To control the deleterious performance of ROS, some bacteria are able to produce enzymes called superoxide dismutase (SOD) that metabolize O_2_ and avoid the harmful cascade of ROS (Imlay 2008). In the culture supernatant of *Mycobacterium tuberculosis,* SOD was identified in high quantity demonstrating its importance in oxidative control (Andersen et al. 1991). Similarly to *M. tuberculosis*, MAP is able to secrete SOD into the culture supernatant, suggesting that pathogenic mycobacteria exhibit this property (Liu et al. 2001). Furthermore, Gram-negative and Gram-positive bacteria have been reported to release ATP to the culture supernatant and their levels are regulated by the growth phase in all bacterial species. Live bacteria appear to deplete extracellular ATP, mainly in the log phase, hydrolyzing or degrading extracellular ATP on the cell surface. Possibly, the extracellular ATP produced by one group of bacteria could provide energy for the growth of another group of bacteria (Mempin et al. 2013). Thus, contrary to what we hypothesize, MAP supernatant stimulates *E. coli* growth and does not interfere with the invasion process *in vitro*. Although the focus of the present study was not research aimed at SOD or extracellular ATP produced by MAP and the growth capacity of *E. coli*, future studies should be performed to clarify this finding.

## 5. Conclusions

Previously we evaluated the influence of *E. coli* on subsequent MAP infection *in vitro*. In the present work we evaluated the influence of MAP on subsequent E. coli infection. The results from the present study demonstrate that the internalization of *E. coli* to bovine mammary epithelial cells previously exposed/infected by MAP was significantly reduced and the translocation through the cells did not alter its efficiency. In general, it may be suggested that the persistence of *E. coli* inside of the bovine mammary gland would be less frequent in MAP-positive cows, either by inhibition of invasion or by rapid translocation to the subepithelial layer. In practical terms, the presence of MAP within mammary epithelial cells could inhibit *E. coli* infection, but once inside the cell, *E. coli* is rapidly translocated to the subepithelial region. In addition, it allows the understanding of pathophysiological mechanisms of bacteria released by milk with importance also in intestinal diseases in humans. To the authors’ knowledge, this is the first study relating the influence of MAP upon *E. coli* invasion on bovine mammary epithelial cells.
